# The insulin polymorphism -23Hph increases the risk for type 1 diabetes mellitus in the Romanian population

**DOI:** 10.1590/S1415-47572010005000074

**Published:** 2010-12-01

**Authors:** Danut Cimponeriu, Pompilia Apostol, Irina Radu, Anne Marie Craciun, Cristian Serafinceanu, Mihai Toma, Cristian Panaite, Dan Cheta

**Affiliations:** 1 Department of Human Genetics and Molecular Diagnosis, Institute of Genetics, University of Bucharest, BucharestRomania; 2 Department of Diabetes, Institute of Diabetes, Nutrition and Metabolic Diseases Nicolae Paulescu, BucharestRomania

**Keywords:** Insulin -23Hph, insulin-like growth factor 2 Apa, diabetes, obesity, case-control study

## Abstract

The insulin -23Hph and IGF2 Apa polymorphisms were genotyped in Romanian patients with T1DM (n = 204), T2DM (n = 215) or obesity (n = 200) and normoponderal healthy subjects (n = 750). The genotypes of both polymorphisms were distributed in concordance with Hardy-Weinberg equilibrium in all groups. The -23Hph AA genotype increased the risk for T1DM (OR: 3.22, 95%CI: 2.09-4.98, p < 0,0001), especially in patients without macroalbuminuria (OR: 4.32, 95%CI: 2.54-7.45, p < 0,0001). No other significant association between the alleles or genotypes of insulin -23Hph and IGF2 Apa and diabetes or obesity was identified.

## Introduction

Insulin and IGF2 (11p15.5) are candidate genes for complex traits, including type I diabetes mellitus (T1DM) (Owerbach and Gabbay, 1994), type II diabetes mellitus (T2DM) (Huxtable *et al.*, 2000) and obesity with onset especially in childhood and middle-age (O'Dell *et al.*, 1999; Le Stunff *et al.*, 2001; Gu *et al.*, 2002; Roth *et al.*, 2002; Heude *et al.*, 2004). It is very difficult to determine the real marker(s) for susceptibility to these complex traits, because many polymorphisms from this region of chromosome 11 are in strong linkage disequilibrium with each other. As a consequence, several functional polymorphisms from the insulin – IGF2 region have been frequently implicated with diabetes and/or obesity in association or linkage based studies. One of these is a VNTR polymorphism located in the insulin promoter, 596 bp upstream of the insulin start codon. Some of its variants may adopt noncanonical conformations that can be recognized by *trans* regulator factors, like Pur-1/MAZ. The Insulin -23HphI and IGF2 Apa are other polymorphisms that have recently received attention (Barratt *et al.*, 2004; Marchand and Polychronakos, 2007), because they may interfere with gene expression (Vafiadis *et al.*, 1998; Kralovicova *et al.*, 2006) and predisposition to some pathologic traits (O'Dell *et al.*, 1997, 1999; Gaunt *et al.*, 2001).

In this study we tested the possible association between two polymorphisms from the insulin – IGF2 region and T1DM, T2DM and obesity.

## Subjects and Methods 

###  Subjects

A total of 1369 unrelated Romanian Caucasians (682 men, 687 women), older than 18 years, was enrolled in the study. Patients with T1DM (n = 204), T2DM (n = 215) or obesity (n = 200) diagnosed for at least two years were selected from the N Paulescu Institute (Bucharest, Romania). T1DM patients were selected if they were younger than 20 years at the onset of the disease, required daily insulin treatment and had a BMI between 18.5-25 kg/m^2^. T2DM patients were included if they had had more than two fasting glycemia values above 140 mg/dL after the age of 30 years old, a BMI of 18.5-30 kg/m^2^ and required no treatment with insulin. Obese patients were selected based on BMI values between 30-40 kg/m^2^ and glycemia in a fasting state ≤ 110 mg/dL. The presence or absence of hypertension (blood pressure above 130/85 mm Hg or treatment with antihypertensive medication), smoking (more than five cigarettes per day, for at least one year) and drinking (at least 25 g of alcohol per day, for at least one year) habits were recorded for all participants.

The control subjects comprised a total of 750 individuals (381 men, 369 women) selected from visitors attending two medical clinics from Bucharest for routine checking. They were matched for age, sex, ethnicity and birth place with patients and were equally distributed into Hc1, Hc2 and Hc3 groups. All normal individuals had no relatives with DM, obesity or other genetic syndromes.

Informed consent was obtained from all subjects before enrolment. The design of the study was in accordance with internationally agreed ethical standards (Declaration of Helsinki, 1964) and approved by the N Paulescu hospital ethical committee.

###  Laboratory procedures

Fasting plasma glucose, serum total cholesterol, serum triglycerides and HbA1C levels were measured using standard laboratory protocols. Genomic DNA was isolated using a commercial kit (Wizard Genomic DNA Purification Kit, Promega) from venous blood (1 mL) collected into tubes containing EDTA. Genotyping for insulin -23Hph (-23 A/T, rs689) and IGF2 Apa (820 G/A*,* rs680) was based on a RFLP method. The INSF 5'tccaggacaggctgcatcag3', INSR 5'agcaatgggcggttggctca3' and IGF2F 5'cttggactttgagtcaaattgg3', IGF2R 5'cctcctttggtcttactggg3' primer pairs (Sigma Genosys) were used for PCR (Corbett Research thermocycler). Amplicons (5 μL) were electrophoresed on agarose gel (2%) after digestion with 6 U of *Hph*I or *Apa*I endonucleases (New England BioLabs). Two researchers who did not know the clinical data of the subjects independently scored all genotypes. Ten percent of randomly selected samples were re-genotyped as a quality control. No errors in genotyping were observed.

###  Statistical analysis

After checking the normality of distribution for continuous variables by means of the Shapiro-Wilk W test, comparisons of these parameters between groups were performed through Mann-Whitney U non-parametric tests. Categorical variables between groups were compared through chi-squared tests, that were also used for verifying genotype departures from Hardy-Weinberg equilibrium within each group and for comparing genotype frequencies between affected and control subjects. Odds ratio (ORs) and their corresponding 95% confidence intervals were calculated for estimating disease risks corresponding to genotypes AA from -23Hph and IGF2Apa*.* All statistical analyses and procedures were performed using commercial software (STATSDirect version 2.6.4).

## Results

The main characteristics of the patients and controls are presented in Table 1. Patients with T2DM (52.41 ± 7.21) were older than those with T1DM (33.01 ± 6.23, p < 0.0001) or obesity (43.39 ± 11.16, p < 0.0001). The BMI and triglycerides levels were higher in T2DM and obese patients compared with Hc2 and Hc3 controls (p < 0.0001). The T2DM patients are more often smokers than control subjects (p = 0.0001). Macroalbuminuria was significantly (Yate's corrected chi2 = 4.54, p = 0.03) more frequent in T1DM (28.92%, M/F: 27/32) than in T2DM (19.53%, M/F: 19/23). The occurrence of hypertension was similar in all groups of patients (~50%).

The distribution of the -23Hph and IGF2 Apa genotypes were in Hardy-Weinberg equilibrium in all groups (Table 2). The Insulin -23Hph (chi^2^ = 0.84, DF = 4, p = 0.93) and IGF2 Apa (chi^2^ = 5.78, DF = 4, p = 0.22) genotypes are similar in the control lots, Hc1, Hc2 and Hc3. We observed a different distribution of -23Hph genotypes in T1DM and Hc1 lots. The -23Hph AA genotype is significantly associated with T1DM (OR: 3.22, 95% CI: 2.09-4.98, p < 0.0001). This genotype remains also more frequent in T1DM than in controls (89.66% *vs.* 50.26%) if patients with macroalbuminuria and their matched controls were excluded from the analysis. In this case, the association between AA genotype and T1DM increases (OR: 4.32, 95% CI: 2.54-7.45, p < 0.0001). The disease risk remains non-significant if the T2DM patients with macroalbuminuria and their matched controls were excluded from analysis.

We did not observe any other significant associations between investigated markers and clinical and biochemical data (*e.g.* age of disease onset, sex).

## Discussion

This is the first study to simultaneously assess the relationship between insulin – IGF2 polymorphisms and T1DM, T2DM and obesity in the Romanian population. Previous studies in other countries have shown that polymorphisms from the insulin – IGF2 region can predispose to some complex pathological traits, particularly those involving diabetes. Several functional polymorphisms or an extended haplotype may represent the genetic bases for these observations. We conducted a case-control study to investigate the relationship between the insulin -23Hph and IGF2 Apa polymorphisms and three common pathological phenotypes: T1DM, T2DM and obesity. Association studies can identify substantial genetic effects (*e.g.* OR > 2.0) between normal and clinical phenotypes using relatively small sample sizes (n = 200) (Dupont and Plummer, 1990). The subjects were selected from an ethnically homogenous population surrounding Bucharest to avoid spurious associations.

The variants of Insulin -23Hph (-23Hph A: 73.53%) and IGF2 Apa (IGF2 A: 77.3%) polymorphisms in our healthy controls are similar to those reported for Caucasians (Bennett and Todd, 1996).

We found a strong association between insulin -23Hph polymorphism and T1DM (OR_AA_ = OR: 3.22, 95% CI: 2.09-4.98, p < 0.0001). This result is in agreement with previous studies based on Caucasian cohorts (Undlien *et al.*, 1994; Krokowski *et al.*, 1997) or families (Davies *et al.*, 1994) which also showed the importance of markers from this region regarding diabetes susceptibility. As a common observation the results of these studies support the importance of markers in linkage disequilibrium with class I VNTR disequilibrium on the risk for T1DM.

In our study 59 patients with T1DM and 37 patients with T2DM have proteinuria. The frequency of the AA genotype in T1DM lot (81.37% *vs.* 50.26%) and the disease risk (OR_AA_: 4.32, 95% CI: 2.54-7.45) increases when patients with macroalbuminuria and their matched controls were excluded from analyses. We did not identify a similar effect in patients with T2DM. The -23Hph A allele is in almost complete linkage disequilibrium with the INS VNTR class I. It has been previously reported that the prevalence of proteinuria in insulin-requiring diabetic patients is significantly higher in homozygotes for INS VNTR class I variants than in carriers of other genotypes (Raffel *et al.*, 1991). Additional studies are necessary to clarify these discordant results.

The genetic background (Wilkin, 2009), body mass (Kibirige *et al.*., 2003; Betts *et al.*, 2005; Knerr *et al.*, 2005), different degree of immune activation (Hathout *et al.*., 2001; Umpaichitra *et al.*, 2002) and insulin resistance seem to represent some common reference points in the complex relationships between T1DM, T2DM and obesity. For this study, we carefully selected nonobese diabetic patients and nondiabetic obese patients in an attempt to reduce the effect of factors which may link these phenotypes. The Insulin -23Hph polymorphism was similarly distributed in T2DM and Hc2 lots (p > 0.05) irrespective of the degree of renal involvement. Similar results have been reported in some (*e.g.* Pima Indians), but not all populations (Ong *et al.*, 1999; Lindsay *et al.*, 2003).

We did not observe a significant association between -23Hph genotypes and obesity in middle aged patients (p > 0.05). This result is in agreement with other studies, in which IDDM2 markers were also found to not change the risk for obesity in middle-aged subjects (Sandhu *et al.*, 2005). A similar distribution of IGF2 Apa in patients and controls was observed in our lots. Although the IGF2 polymorphisms were also not associated with BMI in middle-aged subjects in other population (Heude *et al.*, 2007), it is speculated that an extended haplotype may better describe the relationship between obesity and response to glucose at least in older men (Rodríguez *et al.*, 2006). In our study the results were non-significant even when the gender was taken into account (p > 0.05).

A predisposition for diabetes and obesity may be triggered by genetic and environmental determinants of growth patterns from prenatal to childhood life (Stene *et al.*, 2001; EURODIAB Substudy 2 Study Group, 2002). We cannot entirely exclude the insulin and IGF2 genes as candidates for these complex traits because data regarding the origin of risk alleles, birth size and postnatal growth development were not available for this study. The contribution of polymorphisms from this region to predisposition for human pathology will be much better understood after testing of additional genetic (*e.g.* cis or trans factors, epigenetic modifications) and non-genetic (*e.g.* maternal factors, intracellular environment) factors.

## Figures and Tables

**Table 1:** Clinical and biochemical data of subjects selected for this study.

Parameters	T1DM	Hc1	T2DM	Hc2	Ob	Hc3
Number	204	250	215	250	200	250
Gender (M/F) (^c^)	105/99	142/108	114/101	135/115	82/118	104/146
Age (in years): Mean ± SD (range)	33.01 ± 6.23 (21-53)	33.4 ± 6.42 (21-53)	52.41 ± 7.21 (31-65)	52.58 ± 7.04 (31-65)	43.39 ± 11.16 (20-64)	44.3 ± 11.32 (20-64)
p (patients *vs.* Hc)^mw^	0.55		0.83		0.4	
Current BMI (kg/m^2^) M ± SD (range)	23.03 ± 1.42 (18.9-24.98)	23.12 ± 1.28 (18.98-24.95)	24.78 ± 2.37 (19.35-29.98)	23.00 ± 1.4 (18.98-24.95)	34.87 ± 2.33 (30.03-39.9)	22.99 ± 1.44 (18.9-24.98)
p (patients *vs.* Hc)^mw^	0.73		< 0.0001		< 0.0001	
HbA1C (%)	8.6 ± 1.8	nd	8.1 ± 2.1	nd	4.8 ± 0.4	nd
Fasting plasma glucose (mg/dL)	nd	88 ± 9	110 ± 15	86 ± 11	92 ± 8	84 ± 10
Total cholesterol ± SD (mg/dL)	179.2 ± 43.3	157.3 ± 31.4	206.8 ± 39.5	138.6 ± 25.2	217.4 ± 50.2	165.1 ± 37.4
Triglycerides ± SD (mg/dL)	112.1 ± 24.84 (62-210)	109.96 ± 16.73 (61-157)	121.71 ± 20.63 (75-211)	106.6 ± 19.92 (61-196)	159.62 ± 69.7 (53-484)	109.82 ± 22.42 (65-198)
p (patients *vs.* Hc)^mw^	0.9501		< 0.0001		< 0.0001	
Smokers (^c^)	25	31	57 p < 0.0001	21	21	26
Drinkers (^c^)	23	29	31	24	26	35

**Table 2:** Genotypes and alleles distributions of the examined polymorphisms in each studied group.

Genotype	T1DM	Hc1	T2DM	Hc2	Ob	Hc3
-23Hph AA	158	129	118	131	107	131
-23Hph AT	40	109	89	104	85	108
-23Hph TT	6	12	8	15	8	11
A%:T%	87.25: 12.75	73.4:26.6	75.58: 24.42	73.2:26.8	74.75:25.25	74:26
HW	2.86	3.4	3.17	0.91	3.17	3.76
Chi2	32.56, p < 0.0001		1.35, p = 0.509		0.08, p = 0.9607	
OR_AA_ (95%CI)	3.22 (2.10-4.98)		1.11 (0.75-1.62)		1.05 (0.71-1.54)	
IGF2 Apa GG	127	139	117	152	115	156
IGF2 Apa GA	69	102	76	85	71	79
IGF2 Apa AA	8	9	22	13	14	15
G%:A%	79.17: 20.83	76: 24	72.1:27.9	77.8:22.2	75.25:24.75	78.2:21.8
HW	0.13	3.51	3.17	0.06	0.44	1.34
Chi2	2.33, p = 0.31		4.76, p = 0.09		1.12, p = 0.57	
OR_AA_ (95%CI)	1.32 (0.89-1.96)		0.77 (0.52-1.13)		0.82 (0.55-1.21)	
